# Transcriptional analysis of *Bemisia tabaci* MEAM1 cryptic species under the selection pressure of neonicotinoids imidacloprid, acetamiprid and thiamethoxam

**DOI:** 10.1186/s12864-021-08241-6

**Published:** 2022-01-05

**Authors:** Cheng Song Zhou, Huan Huan Lv, Xiao Hu Guo, Qian Cao, Rui Xingyue Zhang, De Ying Ma

**Affiliations:** 1 Engineering Research Centre of Cotton, Ministry of Education /College of Agriculture, Xinjiang Agricultural University, 311 Nongda East Road, Urumqi, 830052 China; 2 Key Laboratory of the Pest Monitoring and Safety Control of Crops and Forests of the Universities of the Xinjiang Uygur Autonomous Region, 311 Nongda East Road, Urumqi, 830052 China; 3 Agricultural Product Inspection and Test Center, 99 Wuyi East Road, Changji, 831100 China

**Keywords:** Neonicotinoids, *Bemisia tabaci* transcriptome, Resistance

## Abstract

**Background:**

Neonicotinoids are widely applied in the control of the destructive agricultural pest *Bemisia tabaci*, and resistance against these chemicals has become a common, severe problem in the control of whiteflies. To investigate the molecular mechanism underlying resistance against nenonicotinoids in whiteflies, RNA-seq technology was applied, and the variation in the transcriptomic profiles of susceptible whiteflies and whiteflies selected by imidacloprid, acetamiprid and thiamethoxam treatment was characterized.

**Results:**

A total of 90.86 GB of clean sequence data were obtained from the 4 transcriptomes. Among the 16,069 assembled genes, 584, 110 and 147 genes were upregulated in the imidacloprid-selected strain (IMI), acetamiprid-selected strain (ACE), and thiamethoxam (THI)-selected strain, respectively, relative to the susceptible strain. Detoxification-related genes including P450s, cuticle protein genes, GSTs, UGTs and molecular chaperone HSP70s were overexpressed in the selected resistant strains, especially in the IMI strain. Five genes were downregulated in all three selected resistant strains, including 2 UDP-glucuronosyltransferase 2B18-like genes (LOC 109030370 and LOC 109032577).

**Conclusions:**

Ten generations of selection with the three neonicotinoids induced different resistance levels and gene expression profiles, mainly involving cuticle protein and P450 genes, in the three selected resistant whitefly strains. The results provide a reference for research on resistance and cross-resistance against neonicotinoids in *B. tabaci*.

**Supplementary Information:**

The online version contains supplementary material available at 10.1186/s12864-021-08241-6.

## Background

The cotton whitefly *Bemisia tabaci* (Gennadius) (Hemiptera: Aleyrodidae) is one of the most invasive and destructive pests worldwide. It has a wide host range including more than 600 species of agriculture and horticulture plants. *B. tabaci* is a complex of at least 41 discrete species that belong to 11 major genetic groups [1–9]. The *B. tabaci* genome is highly divergent from other sequenced hemipteran genomes, showing no detectable synteny [10]. MEAM1 (Middle East-Asia Minor 1, B biotype) and MED (Mediterranean, Q biotype) are the two most widespread and damaging cryptic species in this complex, and MEAM1 has been called a “superbug” because of its highly destructive power in local agriculture [11]. The first global invasion of whiteflies was caused by the transportation of MEAM1 cryptic species on ornamental plants through trade since the late 1980s [12]. MEAM1 whitefly spread to Xinjiang, China, at the end of the 1990s, and its status has been elevated from an accidental pest to a major pest in a short time; these whiteflies remain in some cotton fields in the southern part of Xinjiang at present. Toxicology tests have shown that the resistance of this pest to several pesticides has increased with intensive chemical application in local areas, especially the application of neonicotinoids [13].

Neonicotinoids are commonly used pesticides that target the nicotinic acetylcholine receptor (nAChR) of the central neural system, resulting in excitation and the convulsion of insects, leading to their to death [14–17]. Imidacloprid, acetamiprid and thiamethoxam are three typical neonicotinoids that were launched in 1991, 1995 and 1998, respectively, and accounted for over 75% of neonicotinoid sales worldwide in 2012 [18]. With the worldwide use of neonicotinoids, whiteflies, especially the MEAM1 and MED cryptic species, have developed different levels of resistance to these insecticides [19–24]. Imidacloprid resistance in whiteflies was first reported in 1996, followed by thiamethoxam and acetamiprid resistance [18, 20, 21, 25].

Increased detoxification enzyme activity and the overexpression of corresponding genes are correlated with the resistance of *B. tabaci* to neonicotine insecticides [26–29]. CYP6CM1 was the first cytochrome P450 reported to be involved in the imidacloprid resistance of the MEAM1 and MED cryptic species based on high overexpression, and it was also found to participate in the metabolism of thiamethoxam, pymetrozine and pyriproxyfen in *B. tabaci* [26, 30–32]. Further research revealed that a mitogen-activated protein kinase (MAPK) signaling pathway mediates the activation of cAMP response element binding protein (CREB) and that CREB mediates the increase in CYP6CM1 expression via phosphorylation-mediated signal transduction [33]. Resistance to imidacloprid in field populations of *B. tabaci* is also associated with the increased expression of CYP6CM1 and another cytochrome p450 gene, CYP4C64 [34]. In addition to cytochrome P450 enzymes, carboxylesterases (COEs), NAD-dependent methanol dehydrogenase and glutathione S-transferases (GSTs) have been suggested to be involved in the thiamethoxam resistance of whiteflies [35–39]. RNA sequencing (RNA-seq) and proteomic research have suggested that the overexpression of GST, UDP glucuronosyltransferase (UGT), glucosyl/glucuronosyl transferase and cytochrome P450 proteins plays a role in resistance against thiamethoxam in *B. tabaci* [40]. CYP303 and CYP6CX3 might be related to the imidacloprid and acetamiprid resistance of *B. tabaci*, and no evidence of nAChR mutation related to the neonicotinoid resistance of *B. tabaci* has been found to date [41].

In this study, we established a susceptible strain of the *B. tabaci* MEAM1 cryptic species by raising the population under controlled indoor conditions without exposure to any pesticide. An imidacloprid-selected strain, acetamiprid-selected strain and thiamethoxam-selected strain were established from the parental susceptible strain by continuous selection with the three neonicotinoids. To determine how this whitefly reacts to long-term selection, the transcriptome profiles of the four strains were sequenced and compared via RNA-seq. It was shown that long-term selection by neonicotinoids led to the upregulation of detoxification-correlated genes belonging to various gene superfamilies (mainly P450 and cuticle proteins), especially in the imidacloprid-selected strain.

## Results

### Resistance levels of the three neonicotinoid-selected strains

After selection by imidacloprid, acetamiprid and thiamethoxam for 10 generations, the three selected neonicotinoid strains originating from the susceptible strain had developed various levels of resistance against the corresponding neonicotinoids in the following order: IMI strain (43.42-fold) > ACE strain (13.63-fold) > THI strain (2.57-fold) (Table 1).

**Table 1:** Resistance level of three resistance-selected strains of MEAM1 cryptic species at the 10th generation

Strains	LC_50_(95% CL)(mg·L-1)	Slope(b ± SE)	RR
IMI	3403.841 (1863.422–4283.085)	2.931 ± 0.688	43.42
ACE	407.727 (264.453–713.769)	0.784 ± 0.164	13.63
THI	340.116 (273.984–432.902)	1.702 ± 0.230	2.57

*IMI* imidacloprid selected strain, *ACE* acetamiprid selected strain, *THI* thiamethoxam selected strain; 95% CL 95% confidence limits, *SE* Standard error, *RR* resistance ratio

### Transcriptome data analysis

RNA-seq was utilized to quantify *B. tabaci* gene expression in the IMI strain, ACE strain, THI strain and SUS strain (control), and a total of 90.86 GB of clean sequence data were obtained. The GC content was 40.07–43.41%, and the Q20 and Q30 values were ≥ 95.81% and ≥ 89.64%, respectively. The average total mapping ratio and average unique mapping ratio were 85.97 and 84.63%, respectively (Table 2).

**Table 2:** Summary of the RNA-seq data

Sample	Raw reads	Clean reads	Clean bases (Gb)	Q20 (%)	Q30 (%)	GC (%)	Total reads	Total map	Unique map
SUS_A	48,504,690	48,490,522	7.27	95.86	90.03	40.07	48,490,522	41,725,916 (86.05%)	41,106,490 (84.77%)
SUS_B	50,196,366	50,181,356	7.53	96.22	90.68	41	50,181,356	43,847,577 (87.38%)	43,175,889 (86.04%)
SUS_C	53,504,154	53,487,908	8.02	96.15	90.6	40.3	53,487,908	46,166,534 (86.31%)	45,443,847 (84.96%)
IMI_A	42,456,440	42,443,694	6.37	96.3	90.88	40.97	42,443,694	37,056,407 (87.31%)	36,520,464 (86.04%)
IMI_B	63,185,144	63,166,110	9.47	96.14	90.42	43.41	63,166,110	56,409,149 (89.3%)	55,417,093 (87.73%)
IMI_C	47,763,844	47,749,700	7.16	96.2	90.74	40.23	47,749,700	41,246,966 (86.38%)	40,629,937 (85.09%)
ACE_A	55,560,384	55,543,710	8.33	96.15	90.59	40.52	55,543,710	47,371,626 (85.29%)	46,617,991 (83.93%)
ACE_B	48,844,738	48,830,222	7.32	96.12	90.51	40.18	48,830,222	41,837,768 (85.68%)	41,197,173 (84.37%)
ACE_C	44,189,186	44,175,744	6.63	96.03	90.38	40.18	44,175,744	37,349,722 (84.55%)	36,757,531 (83.21%)
THI_A	48,398,214	48,383,968	7.26	95.81	89.64	42.38	48,383,968	41,554,580 (85.89%)	40,870,267 (84.47%)
THI_B	53,127,326	53,111,186	7.97	96.08	90.53	40.09	53,111,186	44,531,359 (83.85%)	43,839,726 (82.54%)
THI_C	50,190,878	50,176,074	7.53	96.16	90.59	41.02	50,176,074	41,993,835 (83.69%)	41,330,531 (82.37%)

SUS_A, B, and C: three repeats from susceptible MEAM1 cryptic species raised under control conditions without contact of any pesticide; IMI_A, B and C: three repeats from susceptible MEAM1 cryptic species selected continuously by imidaclorprid for 10 generations; ACE_A, B and C: three repeats from susceptible MEAM1 cryptic species selected continuously by acetamiprid for 10 generations; THI_A, B and C: three repeats from susceptible MEAM1 cryptic species selected continuously by thiamethoxam for 10 generations; Q20: the percentage of bases with a Phred value > 20; Q30: the percentage of bases with a Phred value > 30. Total reads: Number of clean reads after quality control; Total map: Number and percentage of clean reads mapped with reference genome; Unique map: Number and percentage of clean reads mapped with unique locus of reference genome

A total of 16,069 genes were merged and assembled based on all the mapped reads from the four strains using Stringtie (1.3.3b) [42]. The distributions of the expression levels of all the genes were similar among the three selected resistant strains and the susceptible strain (Additional file 1). More than 68% of the genes were overexpressed (FPKM ≥1), and approximately 4.3% of the genes were extremely highly expressed (FPKM > 100) in each strain (Additional file 2).

A total of 1079 new transcripts were detected, and 208 genes corresponded to transcripts in the NCBI RefSeq of *B. tabaci* (ASM185493v1) (Additional file 3). According to Gene Ontology (GO) [43] classifications, 111 genes were assigned to corresponding GO terms (Additional file 4). The Kyoto Encyclopedia of Genes and Genomes (KEGG) enrichment results revealed 43 genes assigned to corresponding pathways, including phagosome, endocytosis, lysosome and metabolic pathways (Additional file 5).

### Differentially expressed genes between the susceptible and selected resistant strains

**Fig. 1:**
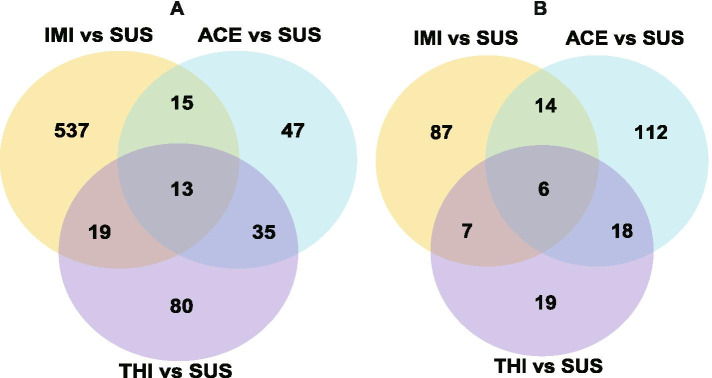
Differentially expressed genes between the whitefly susceptible strain and three resistance selected strains. **A**: Venn plot of up-regulated DEG numbers between the whitefly susceptible strain and three resistance selected strains. **B**: Venn plot of down-regulated DEG numbers between the whitefly susceptible strain and three resistance selected strains. Abbreviations: SUS, susceptible strain; IMI, imidacloprid selected strain; ACE, acetamiprid selected strain; THI, thiamethoxam selected strain

Relative to the SUS strain, 584 genes were upregulated and 114 genes were downregulated in the IMI strain, 110 genes were upregulated and 150 genes were downregulated in the ACE strain, 147 genes were upregulated and 50 genes were downregulated in the THI strain. Thirteen genes were upregulated in all 3 selected resistant strains, and 8 of the 13 genes were “protein coding” biotypes. While 4 of these genes were annotated as E3 ubiquitin-protein ligase BRE1-like transcript variant X1, protein D1-like transcript variant X5, maltase 2-like and zingipain-2-like transcript variant X2, the annotation of the remaining genes will require more database information for reference. Two phase II enzymes, UDP-glucuronosyltransferase 2B18-like genes (LOC109030370 and LOC109032577), were obviously downregulated in all three selected resistant strains relative to the SUS strain (Fig. 1) (Additional files 6, 7, 8).

**Fig. 2:**
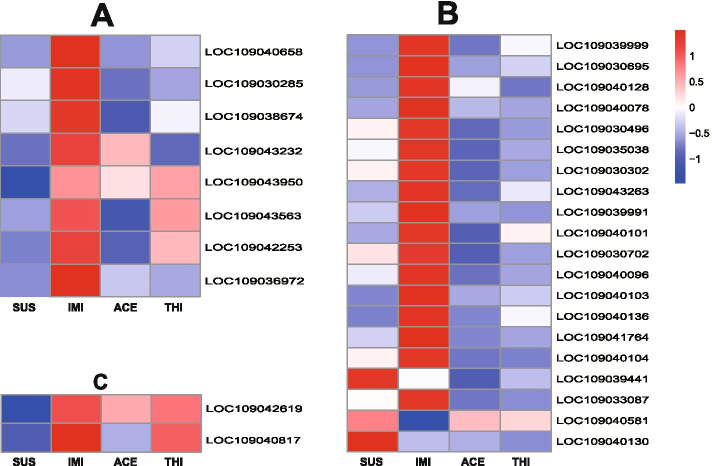
Heatmaps of differentially expressed genes correlated with pesticide resistance. Log2foldchange was calculated by DESeq2 method; Log2foldchange value > 1 or < − 1 with padj value < 0.05 was considered as significant. **A**: Differentially expressed cytochrome P450 genes among the four strains **B**: Differentially expressed cuticle protein genes among the four strains. **C**: Differentially expressed acetylcholine receptor genes among the four strains. Abbreviations: SUS, susceptible strain; IMI, imidacloprid selected strain; ACE, acetamiprid selected strain; THI, thiamethoxam selected strain

The IMI strain exhibited the largest number of upregulated detoxification genes, including 17 cuticle proteins, 8 P450s, 1 carboxylesterase, 1 UDP-glucuronosyltransferase, 1 ABC (ATP-binding cassette), 3 HSPs (heat shock proteins) and 3 potassium channel subfamily K members. Among these genes, venom carboxylesterase-6-like (COE) (LOC109038718) was most highly overexpressed (log2FC = 3.77), followed by cytochrome P450 4C1-like (LOC 109043232) and 10 cuticle proteins (log2FC > 2.00). Cytochrome P450 4C1-like (LOC109043232) and glutathione S-transferase-like transcript variant X1 (LOC109029898) were overexpressed in the ACE strain. Cytochrome P450 4c3-like transcript variant X1 (LOC109043950) and HSP 68-like (LOC109035112) were upregulated in the THI strain (Fig. 2A) (Additional file 9).

A total of 17 cuticle protein genes were overexpressed in the IMI strain, including 4 larval cuticle protein A2B-like genes (LOC 109040103, LOC109040136, LOC 109041764, LOC 109040104) and 1 pupal cuticle protein G1A-like (LOC109033087). Ten of the cuticle protein genes were highly expressed (log2FC > 2). No cuticle protein genes were upregulated in the ACE and THI strains. In contrast, 6 cuticle protein genes, including a pupal cuticle protein G1A-like gene (LOC 109033087, log2FC = − 1.19), were downregulated in the ACE strain. Cuticle protein 8-like (LOC109040130) was inhibited in the THI strain (log2FC = − 6.47) and was also the most obviously reduced gene in the strain (Fig. 2B) (Additional file 10).

A total of 13 acetylcholine receptors were annotated in all of the transcriptome profiles, 2 of which were muscarinic acetylcholine receptors (mAChRs), and 11 of which were nicotinic acetylcholine receptors (nAChRs). Nine of the nAChRs belonged to the α subunit group, and the other 2 belonged to the β subunit group. mAChR (LOC109042619) was upregulated in the IMI strain (log2FC = 1.16) and THI strain (log2FC = 1.06) relative to the SUS strain, and nAChR subunit alpha-L1 (LOC109040817, log2FC = 1.10) was upregulated in the IMI strain. No acetylcholine receptors were found to be differentially expressed between the ACE and SUS groups (Fig. 2C) (Additional file 11).

### GO and KEGG enrichment analysis

**Fig. 3:**
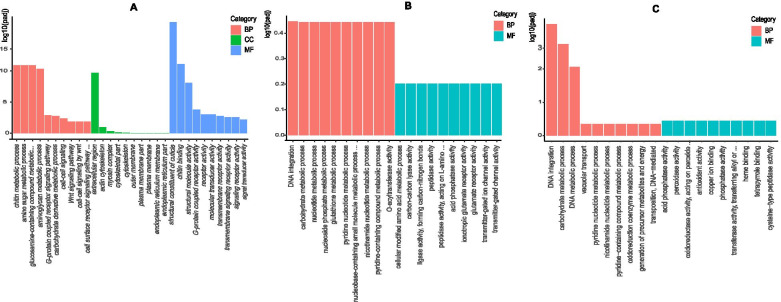
GO enriched terms of DEGs of the three resistance selected strains versus the susceptible strain. The x-axis lists the sub-Go terms under categories of biological process, cellular component, and molecular function. The y-axis is the significant level of GO term enrichment, and a larger value represents a more significant level. **A**: imidacloprid selected strain versus susceptible strain. **B**: acetamiprid selected strain versus susceptible strain. **C**: thiamethoxam selected strain versus susceptible strain. Abbreviations: BP, biological process; CC, cellular component; MF, molecular function

GO enrichment was carried out according to a threshold of padj < 0.05 to analyze the upregulated differentially expressed genes (DEGs) between the SUS strain and the IMI, ACE and THI strains. Compared with the SUS strain, upregulated DEGs in the IMI strain were mainly enriched in biological process (BP) categories including the chitin metabolic, amino sugar metabolic, and glucosamine-containing compound metabolic categories molecular function (MF) categories including the structural constituent of cuticle, chitin binding, and structural molecule activity categories; only 19 genes were enriched in cellular component (CC) categories, among which extracellular region was the predominant in the category. The upregulated genes in the ACE strain were enriched in BP and MF categories, among which DNA integration and O-acyltransferase activity were the two predominant categories, and no genes were enriched in any CC category. The upregulated genes in the THI strain were also enriched in the BP and MF categories, among which DNA integration and acid phosphatase activity were the two predominant categories, and no genes were enriched in any CC category (Fig. 3).

**Fig. 4:**
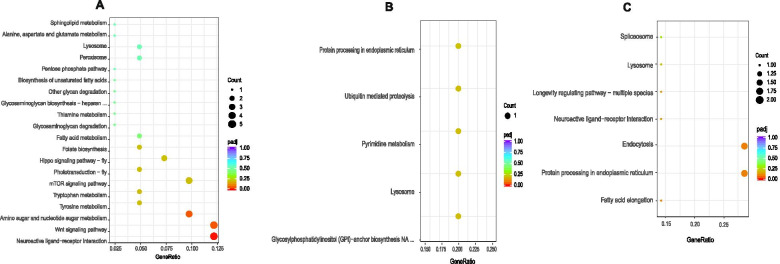
KEGG enriched pathways in the resistance selected strains versus the susceptible strain. The pathways were deduced by pairwise comparison of DEGs between the resistance selected strains versus the susceptible strain. Gene ratio represents the ratio of DEG numbers versus the number of genes annotated in the pathway. Larger gene ratio indicates a greater level of enrichment. The padj values ranging from 0 to 1 are *P* values corrected by multiple hypothesis tests, with lower values indicating greater enrichment. **A**: imidacloprid selected strain versus susceptible strain. **B**: acetamiprid selected strain versus susceptible strain. **C**: thiamethoxam selected strain versus susceptible strain

KEGG pathway enrichment analysis was performed using the Cluster Profiler R package. Neuroactive ligand-receptor interaction and the Wnt signaling pathway were the two most significantly DEG-enriched pathways (false discovery rate < 0.05) between the IMI and SUS strains (Fig. 4A). Two overrepresented pathways, lysosome and pentose and glucuronate interconversions, were identified among the DEGs between the ACE strain and SUS strain (Fig. 4B). Fatty acid elongation and protein processing in the endoplasmic reticulum were the two most significantly enriched DEGs between the THI strain and the SUS strain (Fig. 4C). The lysosome pathway was found to be significantly enriched in all 3 selected resistant strains.

### Validation of DEGs by qRT-PCR

**Fig. 5:**
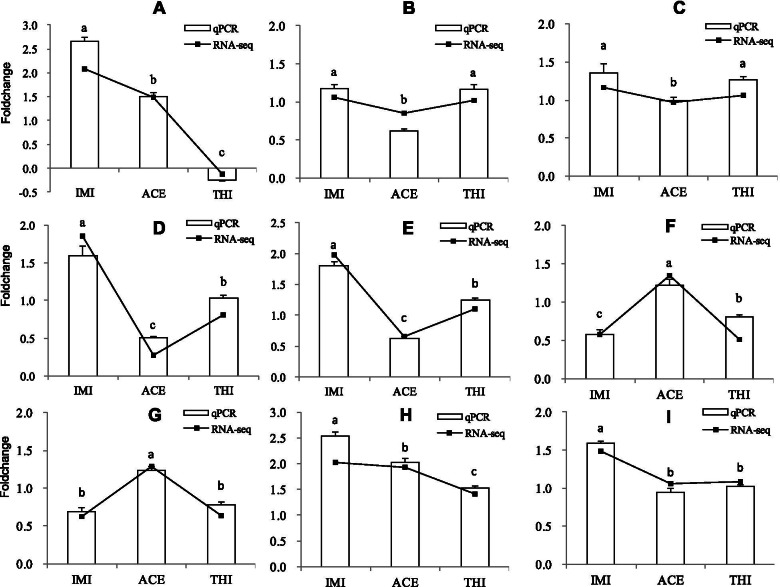
Validation of gene expression patterns by RT-qPCR. Means (± SE) were used to determine transcript levels with the 2^-ΔΔCt^ method. One-way analysis of variance (ANOVA) was used to analyze the relative expression levels of selected genes. Panels **A** - **I**: cytochrome P450 4C1-like; cytochrome P450 4C3-like transcript variant X1; probable muscarinic acetylcholine receptor gar-2 transcript variant X1; heat shock protein 68-like (LOC109040186); heat shock protein 68-like (LOC109035112); glutathione S-transferase-like transcript variant X1; cathepsin B-like; zingipain-2-like transcript variant X2; maltase 2-like. Columns labeled with different lowercase letters indicate significant differences among the three resistance selected strains. Turkey’s multiple range test was used for pairwise comparison for mean separation (*P* < 0.05). The RNA-seq results were represented by broken lines and RT-qPCR results were represented by columns

The expression levels of nine genes in the three resistant selected strains were compared by real-time quantitative PCR, and the expression patterns were consistent with the RNA-seq results (Fig. 5). Three kinds of genes were selected to check the consistency of the gene expression trends (selected resistant strains versus susceptible strains) between the RNA-seq and qPCR results. The first group of genes were upregulated (log2FC > 1) in all the selected resistant strains and included genes such as zingipain-2-like transcript variant X2 and maltase 2-like. The second group of genes were upregulated in one or two selected resistant strains relative to the susceptible strain, which included genes such as P450 4C1-like, probable muscarinic acetylcholine receptor gar-2 transcript variant X1, heat shock protein 68-like (LOC109040186), heat shock protein 68-like (LOC109035112), glutathione S-transferase-like transcript variant X1, and cathepsin B-like. Cytochrome P450 4C3-like transcript variant X1 belonged to the third group of genes, which were not upregulated in any of the selected resistant strains relative to the susceptible strain.

## Discussion

Resistance against imidacloprid has become a common and severe problem among whitefly populations worldwide. In this study, the susceptible MEAM1 whitefly population developed the highest resistance level against imidacloprid (RR = 43.42) after 10 generations of selection relative to acetamiprid selection (RR = 13.63) and thiamethoxam selection (RR = 2.57) under laboratory conditions. Whitefly seems to be able to develop a high level of resistance against imidacloprid within several generations, which might lead to the variation in cross-resistance among neonicotinoids. After continuous selection with thiamethoxam for 30 generations (B biotype), the laboratory strain TH-R (resistance ratio = 25.6) developed a higher level of cross-resistance against imidacloprid (cross-resistance ratio = 47.3) than acetamiprid (cross-resistance ratio = 35.8) [35]. Q biotype whiteflies collected in 2000 (Spain) and 2001 (Germany) showed the highest resistance against imidacloprid, and neonicotinoid cross-resistance decreased in the order of imidacloprid > thiamethoxam > acetamiprid [44]. The physiological and molecular mechanisms underlying the ability to rapidly establish resistance against imidacloprid are worth considering.

Physiological research has revealed that pesticide resistance is largely associated with increased detoxification enzyme activity, and the P450 enzyme is crucial for the establishment of resistance against neonicotinoids [35, 44, 45]. Important molecular evidence of neonicotinoid resistance was also obtained, and CYP6CM1 was suggested to be one of the most important genes in imidacloprid resistance in whiteflies [26, 46, 47]. In this study, transcriptomic comparisons between the three neonicotinoid-selected MEAM1 whitefly strains and the susceptible strain revealed that continuous selection by imidacloprid led to the overexpression of a greater number of genes (especially P450 and cuticle protein genes) than acetamiprid selection or thiamethoxam selection. Despite breakthroughs in imidacloprid resistance research in whiteflies, key genes involved in the metabolism of thiamethoxam and acetamiprid remain unclear. In the transcriptome of the IMI strain, eight P450s were obviously upregulated, including CYP6CM1 and four P450 4C1-like genes, while the P450 4C1-like gene (LOC109043232) was the only overexpressed P450 superfamily member in the ACE strain transcriptome, and the P450 4C3-like gene (LOC 109043950) was the only overexpressed P450 in the THI strain transcriptome. Further study of these P450s might provide us with a comprehensive view of the survival strategy and neonicotinoid resistance formation in the whitefly MEAM1 cryptic species.

Insect cuticle is a crucial determinant of insecticide resistance, and cuticle modifications are mainly attributed to the overexpression of cuticular protein genes [48]. In vivo penetration assays using [^3^ H] imidacloprid revealed a significant reduction in the penetration of the insecticide through the cuticle in a resistant clone of the peach-potato aphid *Myzus persicae* [49]. In the current study, the large number and high levels of overexpressed cuticle proteins in the IMI strain obviously increased resistance against the pesticide. Cuticle protein 8, together with 12 other cuticle protein genes, was significantly downregulated in a thiamethoxam-resistant strain of the cotton aphid *Aphis gossypii* Glover [50]; similarly, cuticle protein 8 was downregulated in the THI strain of whiteflies in our study. How the up- or downregulation pattern of cuticle protein genes affects resistance against the three tested neonicotinoids warrants further investigation.

The toxicity of neonicotinoids is related to the binding affinity of neonicotinoids and relative nAChRs to some extent [14, 51, 52]. An RNA-seq analysis of MED whiteflies revealed no nAChR polymorphism potentially related to resistant phenotypes, but nAChR subunits β2 and α7 were upregulated in an acetamiprid-selected strain and imidacloprid-selected strain relative to a susceptible strain [41]. In the current study, nAChRα-L1 was obviously upregulated in the IMI strain and slightly upregulated in the THI strain (log2FC = 0.88), and nAChR α3 (log2FC = 0.93) and nAChR β1 (log2FC = 0.72) were also upregulated in the IMI strain. Unlike the common link between the overexpression of dextoxification enzyme genes and pesticide resistance, decreased expression levels of nAChR subunits β1 and β2 were observed after imidacloprid exposure in *Apis cerana cerana* [53], and reduced expression of nAChR subunit α2 was correlated with the neonicotinoid resistance phenotype in *Musca domestica* L. [54]. The whiteflies used in both the research by Illias et al. (2015) and our study included newly emerged adult whiteflies without further pesticide treatment, which showed the upregulation of some nAChRs, while the expression of nAChRs in insects that had experienced recent neonicotinoid treatment was obviously reduced, as mentioned above. The exposure of the larval aphid *Acyrthosiphon pisum* to imidacloprid and thiamethoxam was shown to result in the up- or downregulation of various nAChRs [55]. It can still be argued that reduced expression of nAChRs is more likely to be correlated with instant neonicotinoid stimuli, and the upregulation of nAChRs is probably a survival strategy implemented by insect pests when experiencing long-term selection by neonicotinoids. A comparison of the expression patterns of nAChRs in selected resistant and susceptible strains of *B. tabaci* before and after neonicotinoid stimulation might provide a more vivid illustration of pesticide resistance mechanisms.

The muscarinic acetylcholine receptor (mAChR) is a metabotropic G-protein-coupled receptor that initiates continuous intracellular signaling events [56]. Five mAChRs exist in mammals (named m1 – m5), which can be fully activated by the classical agonist muscarine and blocked by the classical mAChR antagonist atropine [57]. GAR-1, GAR-2 and GAR-3 are three new mAChRs in *Caenorhabditis elegans* that are similar to but pharmacologically distinct from mAChRs m1- m5 [58, 59]. GAR-2 is most similar to GAR-1 and closely related to GAR-3, but GAR-2 was not inhibited at any of three tested muscarinic antagonists (atropine, scopolamine, and pirenzepine) and was found to mainly be expressed in different types of neuronal cells [58]. In our study, mAChR gar-2 was upregulated in all three neonicotinoid-selected strains, and mAChR DM1 was slightly upregulated in the IMI strain. Since no insect mAChR has been reported to participate in the establishment of insecticide resistance, whether the observed overexpression is related to the resistance of the whitefly MEAM1 cryptic species needs further examination.

GSTs and UGTs are thought to be involved in the metabolism of xenobiotics in phase II by increasing the polarity of xenobiotics and facilitating their excretion. GSTd7 has been related to resistance to imidacloprid in the MEAM1 and MED cryptic species [60], and GST14 is thought to be related to the resistance of thiamethoxam of the Q-biotype [61]. UGTs have been connected with resistance against imidacloprid, thiamethoxam, and chlorantraniliprole in *Leptinotarsa decemlineata*, *Aphis gossypii Glover*, and *Plutella xylostella* (L.), respectively [62–66]. In the current study, long-term selection with the three neonicotinoids led to the overexpression of one GST gene (glutathione S-transferase-like variant X1, Sigma class) in the ACE strain and one UGT gene (UGT 2B7-like, 2B class) in the IMI strain. No GSTs were found to be up- or downregulated in the IMI strain or the THI strain. Since no GSTs or UGTs of the same classes as the two differentially expressed GST-like and UGT 2B-like genes have been associated with pesticide resistance in whiteflies or other insect pests, how these two genes are related to imidacloprid resistance or acetamiprid resistance needs further investigation.

## Conclusions

Whiteflies of the MEAM1 cryptic species originating from the susceptible parental strain were selected continuously for 10 generations with imidacloprid, acetamiprid or thiamethoxam. The transcriptomes of the four examined strains were established by RNA-seq to investigate the resistance mechanism and survival strategy under the selection pressure imposed by neonicotinoids. The results demonstrated that different resistance levels developed in the whitefly MEAM1 cryptic species in the order of IMI > ACE > THI. In accord with the significantly higher resistance level, more DEGs were found in the IMI strain, mainly P450s and cuticle proteins. DEGs were also found in the ACE strain and THI strain, including P450s, GSTs, nAChRs, mAChRs, HSPs, and COEs. The DEGs between the susceptible strain and the selected resistant strains will provide a basis for further understanding neonicotinoid resistance and cross-resistance in *B. tabaci.*

## Materials and methods

### Insects

The susceptible MEAM1 (SUS) strain was collected at Xinjiang Agricultural University in 2010 and reared indoors on cotton plants without any exposure to insecticides thereafter. The identity of the strain was determined by mitochondrial cytochrome oxidase I gene sequencing (mtCOI) [67].

### Resistance selection

Adults of the susceptible strain were treated with imidacloprid, acetamiprid and thiamethoxam for 10 generations to establish an imidacloprid-selected (IMI) strain, acetamiprid-selected (ACE) strain and thiamethoxam-selected (THI) strain as follows. The following technical products were used for resistance selection and bioassays: imidacloprid (700 g/kg, Bayer CropScience), acetamiprid (200 g/kg, Nippon Soda), and thiamethoxam (250 g/kg, Syngenta). For resistance selection, the three pesticides were diluted with 0.01% Tween 80 to test the LC_50_ value against the corresponding strain in each generation, and the population was then separately selected with the three pesticides based on the LC_50_ values. The LC_50_ value test was carried out following methods described previously with slight modification [13, 68]. In brief, cotton leaves were cut into discs (38 mm diameter), which were then dipped into pesticide solutions for 20 s and allowed to dry in air. The leaf discs were then transferred to agar (1.7%) plates prepared in advance. Female adults that emerged after 2–3 days were collected and transferred to the treated cotton leaf discs and kept for 48 h; 30–40 individuals were used for each of the treatments, which were replicated 3 times. The LC_50_ values were calculated with the PoloPlus Software Program (Leora Software, Berkeley, CA). The three pesticides were then diluted to the proper concentration based on the LC_50_ value, sprayed on cotton leaves and allowed to dry. Adult whiteflies were then transferred to the treated cotton plants and selected for one generation. The procedure was repeated in each generation until the 10th generation for further testing. All strains were kept under controlled environmental conditions with a temperature of 25 ± 1 °C, relative humidity of 70–880% and a photoperiod of 16 L/8 D.

### RNA extraction and quality control

Total RNA was extracted from susceptible and resistant MEAM1 whitefly adults (mixture of 50 male individuals and 50 female individuals). Three biological replicates were included for each of the 4 strains, and RNA integrity was assessed using the RNA Nano 6000 Assay Kit of the Bioanalyzer 2100 system (Agilent Technologies, CA, USA).

### Library preparation for sequencing

A total amount of 1 μg RNA per sample was used for RNA sample preparation. Messenger RNA (mRNA) was purified from total RNA using poly-T oligo-attached magnetic beads. Fragmentation was carried out using divalent cations under elevated temperature in First Strand Synthesis Reaction Buffer (5X). First-strand cDNA was synthesized using random hexamer primers and M-MuLV reverse transcriptase (RNase H). Second-strand cDNA synthesis was subsequently performed using DNA polymerase I and RNase H. The remaining overhangs were converted into blunt ends via exon-nuclease/polymerase activities. After the adenylation of the 3′ ends of the DNA fragments, adaptors with hairpin loop structures were ligated to prepare for hybridization. To preferentially select cDNA fragments of 370–420 bp in length, the library fragments were purified with the AMPure XP system (Beckman Coulter, Beverly, USA). Then, PCR was performed with Phusion High-Fidelity DNA polymerase, universal PCR primers and an index (X) Primer. Finally, the PCR products were purified (AMPure XP system), and library quality was assessed on an Agilent Bioanalyzer 2100 system. The clustering of the index-coded samples was performed on a cBot Cluster Generation System using a TruSeq PE Cluster Kit v3-cBot-HS (Illumina) according to the manufacturer’s instructions. After cluster generation, the library preparations were sequenced on an Illumina NovaSeq platform, and 150 bp paired-end reads were generated [69].

### Transcriptome data analysis

Raw data (raw reads) in FASTQ format were first processed with in-house Perl scripts. In this step, clean data (clean reads) were obtained by removing reads containing adapters, reads containing poly-N sequences and low-quality reads from the raw data. At the same time, the Q20, Q30, and GC contents of the clean data were calculated. All downstream analyses were based on clean data with high quality. Reference genome and gene model annotation files were downloaded from the *B. tabaci* reference genome (assembly ASM185493v1, http://www.whiteflygenomics.org/cgi-bin/bta/index.cgi) [10]. The index of the reference genome was built using Hisat2 v2.0.5, and paired-end clean reads were aligned to the reference genome using Hisat2 v2.0.5 [70]. We selected Hisat2 as the mapping tool because Hisat2 can generate a database of splice junctions based on the gene model annotation file and thus provides better mapping results than other non-splice mapping tools.

### Differential gene expression analysis and functional enrichment

Feature Counts v1.5.0-p3 [71] was used to count the read numbers mapped to each gene. Then, the FPKM value (the expected number of fragments per kilobase of transcript sequence per million base pairs sequenced) of each gene was calculated based on the length of the gene and read count mapped to the gene. The differential expression analysis of two conditions/groups (two biological replicates per condition) was performed using the DESeq2 R package (1.20.0) [72, 73]. A corrected *P*-value of 0.05 and a log2 foldchange of ±1 were set as the thresholds for significant differential expression.

### GO annotation and GO/KEGG enrichment

The GO [43] enrichment analysis of DEGs was implemented with the cluster Profiler R package, in which gene length bias was corrected. GO terms with corrected *P* values < 0.05 were considered significantly enriched by DEGs. The Cluster Profiler R package was used to test the statistical enrichment of DEGs in KEGG pathways (http://www.genome.jp/kegg/) [74, 75]. Gene set enrichment analysis (GSEA) is a computational approach for determining whether a predefined gene set shows a significant consistent difference between two biological states. The genes were ranked according to the degree of differential expression in the two samples, and the predefined gene set was then tested to determine whether the enriched genes were at the top or bottom of the list. GSEA can include subtle expression changes. We used the local version of the GSEA tool (http://www.broadinstitute.org/gsea/index.jsp), and the GO and KEGG datasets were used for GSEA independently.

### Validation of DEGs by quantitative real-time PCR (qRT-PCR)

Primers for nine genes were designed and validated by qRT-PCR. The primers (Additional file 12) were designed with Primer3plus (http://www.primer3plus.com/cgi-bin/dev/primer3plus.cgi). First-strand cDNA was synthesized using Total RNA Extractor (TRIzol) (Sangon Biotech, China) and an M-MuLV First Strand cDNA Synthesis Kit (Sangon Biotech, China). Total RNA (0.5 μg) was reverse-transcribed, and qRT-PCR was conducted with a 7500 Fast Real-time PCR System (ABI) in a 20 μl reaction volume using 2X SG Fast qPCR Master Mix (Low Rox) (Sangon Biotech, China) according to the following protocol: 3 min of activation at 95 °C, followed by 40 cycles of 3 s at 95 °C and 25 s at 60 °C. Relative changes in gene expression were assessed using the 2^−ΔΔCt^ method [76], and elongation factor 1 alpha (EF1α) was used as a reference gene [77, 78]. Samples were assessed in triplicate. Data were analyzed by one-way ANOVA, followed by Tukey’s multiple comparisons and analysis with SPSS v. 19.0 (SPSS, Chicago, IL, USA). For ANOVA, the data were transformed for homogeneity of variance tests. Differences were considered statistically significant when *P* < 0.05.

## Supplementary Information

**Additional file 1.**

**Additional file 2.**

**Additional file 3.**

**Additional file 4.**

**Additional file 5.**

**Additional file 6.**

**Additional file 7.**

**Additional file 8.**

**Additional file 9.**

**Additional file 10.**

**Additional file 11.**

**Additional file 12.**

## Data Availability

The RNA-Seq data are available from the NCBI/GenBank BioProject database under the identifier PRJNA752712.
