# Supplementary figures and images for: Transcriptional analysis of Bemisia tabaci MEAM1 cryptic species under the selection pressure of neonicotinoids imidacloprid, acetamiprid and thiamethoxam
Source: BMC Genomics. 2022 Jan 5;23:15. doi: 10.1186/s12864-021-08241-6 (PMC8728913; doi:10.1186/s12864-021-08241-6)

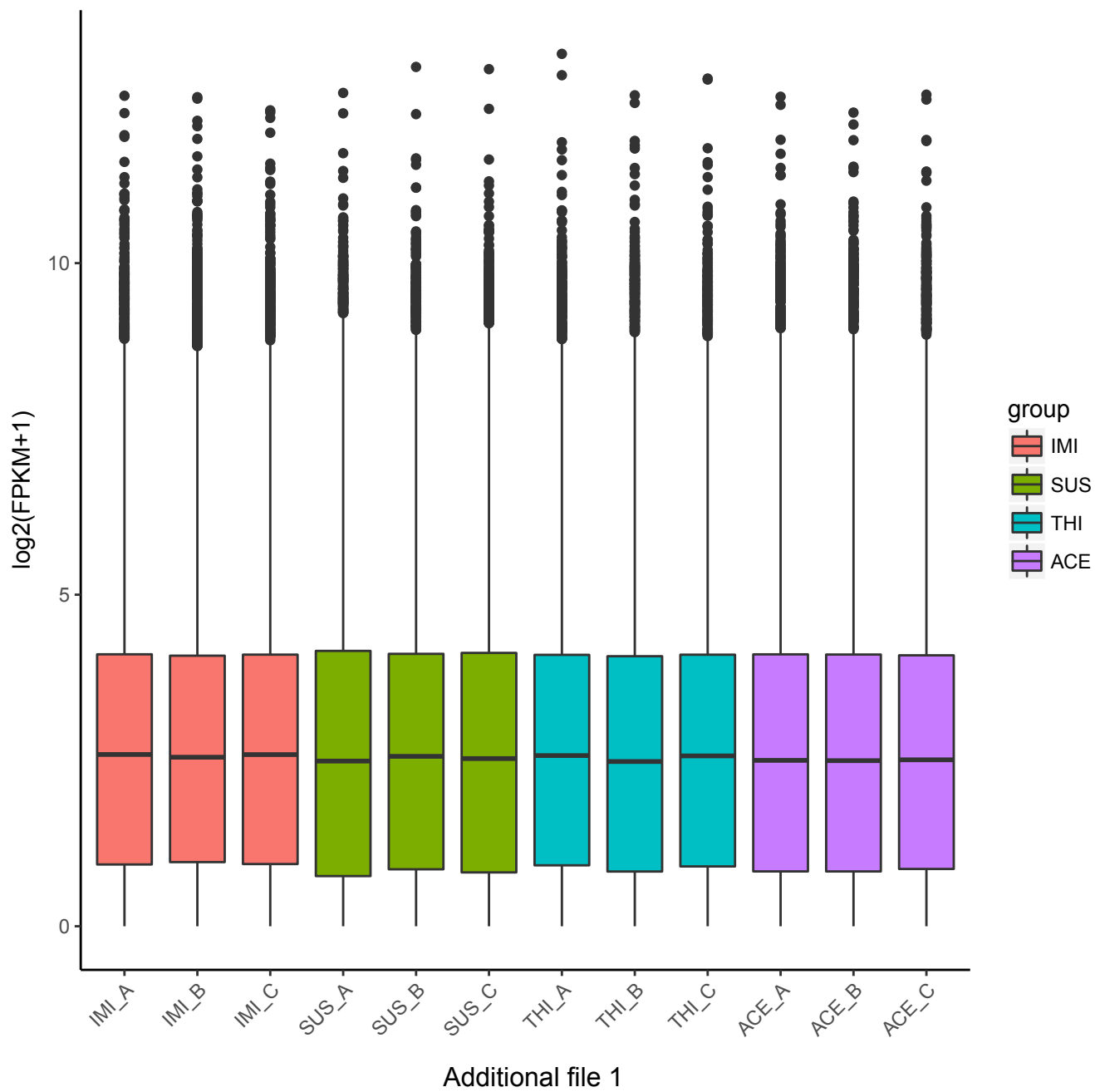

Supplement: Supplementary file 1 — Additional file 1. [file 12864_2021_8241_MOESM1_ESM.pdf]
